# Clinical Observation of Comfort Nursing Combined With Continuous Nursing Intervention After Discharge on Improving Pressure Ulcers, Falls, Quality of Life, and Prognosis in Patients With Intracerebral Hemorrhage

**DOI:** 10.3389/fsurg.2021.829227

**Published:** 2022-02-01

**Authors:** Ji Min Wang, Zhen Liu, Hongxia Liu

**Affiliations:** ^1^Department of Traditional Chinese Medicine, Xiangyang Central Hospital, Affiliated Hospital of Hubei University of Arts and Science, Xiangyang, China; ^2^Department of Neurology, Xiangyang Central Hospital, Affiliated Hospital of Hubei University of Arts and Science, Xiangyang, China

**Keywords:** intracerebral hemorrhage, comfort nursing, continuous nursing, pressure ulcers, falls, quality of life, prognosis

## Abstract

In this prospective study, we randomly divided 131 patients with intracerebral hemorrhage (ICH) who met the inclusion criteria into two groups. One group received routine nursing during hospitalization, and the “Stroke Prevention Knowledge Manual” was issued before discharge, and was recorded as the control group (*n* = 61); one group received comfort nursing during hospitalization, and implemented continuous nursing after discharge, and was recorded as the research group (*n* = 70). The indicators we observed were the occurrence of pressure ulcers and falls during the hospitalization of the two groups of patients and the improvement in neurological function, limb function, quality of life, ability of daily living, and emotional state after the intervention. We also compared the disability degree of the two groups 6 months after discharge, the readmission status within 6 months of discharge, and the nursing satisfaction after the intervention. Our conclusion is that comfort nursing combined with continuous nursing intervention after discharge can effectively reduce the occurrence of pressure ulcers and falls during the nursing period of patients with ICH and contribute to the improvement of their quality of life and prognosis. It is worthy of clinical promotion.

## Introduction

Intracerebral hemorrhage (ICH) is an acute hemorrhagic stroke in which non-traumatic factors cause the rupture of cerebral arteries and the accumulation of blood around the brain tissue, which damages the normal nerve function of the brain, and it accounts for about 10–30% of all patients with stroke (1, 2). The clinical symptoms of the disease are complex and the treatment cycle is long. In addition to the limbs and nerve dysfunction caused by the disease itself, some patients who need to receive long-term bed treatment due to coma, intracranial hypertension, or large bleeding are also prone to pressure sores, pulmonary infection, and other immobile complications, which complicates the condition (3). In addition, falls are also a common adverse event in patients with ICH, and the cause may be related to the decline in cognitive level of patients with ICH and motor and sensory functions (4). For the treatment of ICH, surgical puncture and drainage is the ideal way to remove hematoma, relieve intracranial hypertension, prevent occupying lesions, and reduce brain herniation in a timely manner. However, 75% of patients may still have different degrees of disability after surgery, so a longer rehabilitation period is required. However, patients with prolonged illness and long-term reduced mobility are very prone to anxiety and depression, which affect the rehabilitation process (5, 6). Under the traditional nursing model, the focus of nursing for patients during hospitalization is mostly on the physiological level, and after discharge, patients can only obtain phased knowledge of rehabilitation treatment by returning to the hospital for follow-up visits, so it is difficult to achieve a harmony of physical, mental, and social health. Comfort nursing, as an extension of the connotation of holistic nursing, focuses on the harmonization of comfort and satisfaction at the physical, psychological, social, and spiritual levels compared to traditional care and is an effective care model to help patients achieve a relaxed and comfortable state and eliminate pain and fatigue symptoms, etc. (7). As an out-of-hospital extension of holistic care in the hospital, continuous nursing is a community and family service model that ensures that patients can still enjoy continuous and coordinated rehabilitation care during the recovery period after discharge, and it has the effect of promoting the rehabilitation process and reducing the need for readmission (8). In this study, comfort nursing and continuous nursing were combined in the nursing management of ICH, and its effects on pressure ulcers, falls, quality of life, and prognosis of patients with ICH were observed. The report is as follows.

## Materials and Methods

### General Information

From March 2018 to December 2019, 131 patients with ICH who were admitted to this hospital were randomly divided into the control group (*n* = 61) and the study group (*n* = 70). There was no statistical difference between the two groups in general information such as gender, age, bleeding site, hematoma volume, diabetes mellitus, and caregivers (*p* > 0.05), as shown in Table 1.

**Table 1:** Comparison of two groups of general information [*n* (%), (M±SD)].

**Types**	**Control group** **(*n* = 61)**	**Study group (*n* = 70)**	*****χ***^2^/*t***	***p***
Gender			0.026	0.871
Male	34 (55.74)	40 (57.14)		
Female	27 (44.26)	30 (42.86)		
Age (year old)	64.52 ± 7.86	63.39 ± 7.67	0.831	0.407
Bleeding site			0.297	0.862
Basal ganglia	36 (59.02)	38 (54.28)		
Brain lobe	18 (29.51)	23 (32.86)		
Thalamus	7 (11.47)	9 (12.86)		
Hematoma volume (mL)	48.73 ± 10.29	49.38 ± 9.90	0.368	0.713
Diabetes			0.283	0.595
Yes	10 (16.39)	14 (20.00)		
No	51 (83.61)	56 (80.00)		
Caregiver			0.412	0.814
Spouse	35 (57.38)	38 (54.29)		
Children	18 (29.51)	20 (28.57)		
Other	8 (13.11)	12 (17.14)		

### Inclusion Criteria

The inclusion criteria included the followings: ① patients who met the diagnostic criteria for spontaneous ICH in Guidelines for the Management of Spontaneous Intracerebral Hemorrhage (9); ② age <75 years; ③ first onset; ④ patients with soft channel puncture and drainage to remove hematoma; ⑤ patients who met the requirements for home visits; ⑥ patients who had signed an informed consent.

### Exclusion Criteria

The exclusion criteria included the followings: ① ICH caused by trauma; ② secondary ICH; ③ patients with brainstem hemorrhage or brainstem failure; ④ patients with vascular dementia, Parkinson's disease, and psychosis; ⑤ patients with malignant tumors.

### Nursing Methods

Both groups were treated with minimally invasive soft channel puncture and drainage after admission to the hospital, and vital signs were monitored and maintained after the operation. Basic treatments such as lowering intracranial pressure, brain-protective agents, control of blood pressure and blood sugar, and prevention of complications were given according to the specific conditions of the patients. Based on this, the control group was received with routine nursing intervention during hospitalization, that was, in-hospital health education, guidance on diet, life and medication, oral, respiratory, and psychological care, and after 48 h of stable illness, routine functional training and active and passive activities of the limbs were carried out. The “Knowledge Handbook of Stroke Prevention and Treatment” was distributed before discharge and rehabilitation training was ordered and returned to the hospital for follow-up treatment 6 months after discharge.

On the basis of the above, the research group was received with comfort nursing intervention during hospitalization. Specifically, (1)Comfortable environment: Keep the ward well-ventilated, well-lit, clean and tidy, suitable for room temperature and humidity, place green plants, and use warm color decorations to provide a comfortable hospital environment for the rehabilitation of patients. (2) Comfortable bedtime: Based on the routine bedtime management (3–4 w), combined with evidence-based nursing basis and expert advice, targeted bedtime guidance was implemented to prevent adverse events such as rebleeding and falls. In other words, for those who had basically disappeared with positive signs of the nervous system and symptoms, they could gradually sit up and get out of bed after 5–7 days of bed rest; for those with mild positive signs of the nervous system and no symptoms of intracranial hypertension, they could perform self-care activities such as dressing, sitting up, eating, and washing in bed after 2 weeks of bed rest and gradually got out of bed after half a month; for severe condition of patients with heavy bleeding or intracranial hypertension, they stayed in bed strictly for 3–4 w; and for subarachnoid hemorrhage, they stayed in bed absolutely for 4–6 w. Those who did not listen to discussion should have patiently explained the reasons and consequences. (3) Comfortable posture: Raise the head of the bed 15–30°, use an antidecubitus air mattress, and assist the patient to turn over and knock their back once every 2.5 h. In the treatment of the affected limbs and joints, put a small soft pillow to keep the affected limbs in a functional position and assist the caregiver to massage the bony processes and compressed areas. Wipe the skin with warm water every day, especially to keep the skin clean at the vulva, buttocks and cornea. Put foot boards on the soles of the feet or wore hard shoes to prevent edema, shoulder and back pain, joint deformities, pressure sores, and foot drop; for people with unconsciousness or agitation, guardrails should be added by the bed to prevent falls hurt. (4)Comfortable sleep: Keep the ward quiet, arrange reasonable time for diagnosis, treatment and nursing activities, and create favorable conditions for the patient's normal sleep–wake cycle, which could soak feet in hot water at night, empty your urinary bladder before going to bed, drink hot milk, listen to light music, etc. For those who suffered from insomnia caused by pain, we should deal with symptoms promptly; for those who were accompanied by anxiety and depression, we should strengthen psychological counseling and give more comfort and companionship. If necessary, oral sedative hypnotic drugs were administered. (5) Comfortable defecation: the reasons and importance of defecating in bed needed to be explained to the patient. The bed should be covered with a sheet, kept dry and clean, and changed in a timely manner. When the ward had many people, a screen was set up to shield and give psychological comfort to the patients. When the patients had difficulty in defecating, the researchers could help them defecate by massaging the lower abdomen appropriately. (6) Comfortable infusion: Use intravenous indwelling needles for infusion, when infusion, keep warm and use a thermostat to warm to about 35°C before infusion, appropriate massage during needle insertion to relieve discomfort, during the infusion, observe closely for redness, swelling, and leakage of the infusion limb. (7)Mental comfort: Medical staff should be confident and calm, communicate actively with patients in friendly language, and introduce past successful cases to patients to ease their panic, respect and understand patients, listen patiently to their main complaints, and promptly resolve the psychological problems that cause their unhappiness. (8)Social comfort: understand the patient's family members and social relationships, communicate with the patient's family more often, and do a good job of psychological counseling for them. It was suggested that the patients should be tolerant and understand each other and strive for effective family and social support for the patients as much as possible.

Continuous nursing care during the rehabilitation period after discharge from the hospital was carried out. Specifically, (1) Conduct targeted bedside teaching or video drills before discharge, and print out the key content and precautions of rehabilitation training and prevention of complications and distribute them to patients. The content mainly included the guidance of language and physical function training, the guidance of medication, diet and daily life, the treatment of spastic hemiplegia, and the prevention and treatment of pressure sores, falls, and emotional guidance. (2) Establishing a “patient rehabilitation group” by WeChat to push knowledge about stroke and its prevention and treatment of complications. Conduct video visits to guide patients' rehabilitation skills and guide communication between patients-to-patients. Organize and record the patient's rehabilitation data and answer the patient's rehabilitation questions. (3) Telephone followed-up once for every 2 w to know the patient's rehabilitation progress and to provide targeted rehabilitation guidance. Nursing staff could help patients build confidence in recovery through psychological hints or examples of successful cases. (4) The first home visit was after 1 month of discharge and once for every 3 months thereafter. Based on the degree of completion of the patient's rehabilitation plan, the patient's subsequent rehabilitation goals were analyzed and revised, and on-site rehabilitation drills were implemented until the patient and caregiver fully master them. The results were compared between the two groups 6 months after discharge.

### Observe Indicators

(1) The occurrence of pressure sores and falls during the nursing period of the two groups was recorded. (2) The neurological function of the two groups before nursing and 6 months after discharge was evaluated by the National Institutes of Health Stroke Scale (NIHSS). It contained 11 items. The total score was 42, the higher the score, the more impaired the neurological function. (3) The limb function of the two groups before nursing and 6 months after discharge was assessed by Fugl-Meyer Scale (FMA). It was divided into upper limb motor function scale (33 subitems) and lower limb motor function scale (17 subitems). The total score was 100, the higher the score, the better the limb motor function. (4) The quality of life of the two groups before nursing and 6 months after discharge was assessed by the Stroke Special Quality of Life Scale (SS-QOL). It contained 12 aspects. The total score was 49–245 points, and the score was directly proportional to the quality of life. (5) The daily living ability of the two groups before nursing and 6 months after discharge was evaluated by the Modified Barthel Index (MBI). It contained 10 items. The total score was 100, the higher the score, the better the life skills. (6) The emotional state of the two groups before nursing and 6 months after discharge was evaluated by the Hospital Anxiety Depression Scale (HAD-A/HAD-D). Both are 7 items, which used Likert 3-level scoring method; 0–7 points, 8–10 points, 11–21 points represented the three emotional states of no anxiety/depression, borderline anxiety/depression, and obvious anxiety/depression, respectively. (7) The degree of disability of the two groups 6 months after discharge was assessed by the Glasgow Outcome Scale (GOS). A five-level scoring method was adopted for grading. Among them, Grade I, Grade II, Grade III, Grade IV, and Grade V represented the five disability states of namely death, plant survival, severe disability, moderate disability, and good disability, respectively. Disability rate = (Grade II + III + IV)/total number of cases. The readmissions within 6 months of discharge from the two groups were recorded. (8) The satisfaction of the two groups after nursing was evaluated by the self-made nursing satisfaction questionnaire. It was divided into three levels: very satisfied, basically satisfied, and unsatisfied (85–100 points, 60–84 points, and 0–60 points). The content validity index of this questionnaire was 0.890, the Cronbach's alpha coefficient was 0.896, and the reliability and validity were good. Satisfaction rate = (very satisfied + basically satisfied) number/total number × 100%.

### Statistical Methods

The SPSS 22.0 software was used, the counting data are expressed as (%), the comparison is performed by chi-square test, the measurement data are expressed as (±s), and t analysis is carried out for comparison. *p* < 0.05 indicates that the difference is statistically significant.

## Results

### Comparison of the Incidence of Pressure Ulcers and Falls Between the Two Groups

**Figure 1:**
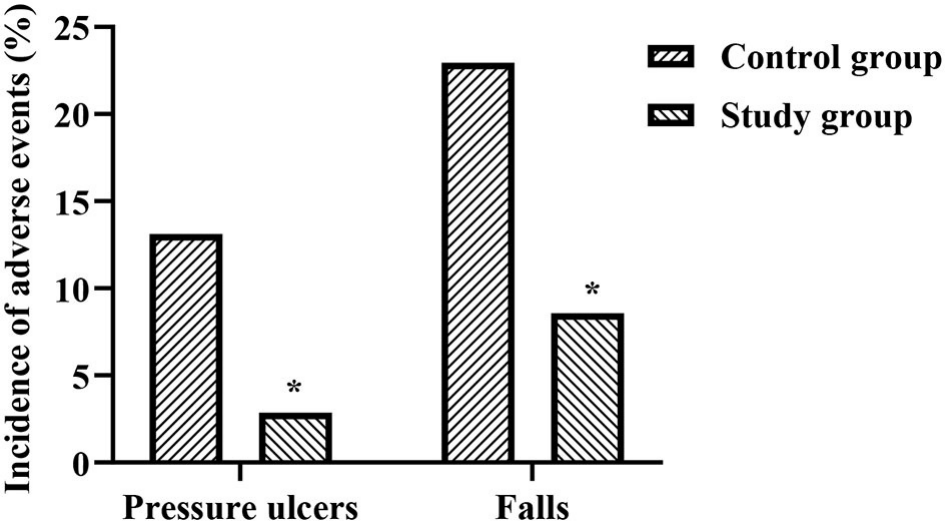
Comparison of the incidence of pressure ulcers and falls between the two groups. *Represents that there was a statistical difference in the control group and the study group.

During the nursing period, the incidence of pressure ulcers in the study group (2.86%) was significantly lower than that in the control group (13.11%) (*p* < 0.05), and the incidence of falls in the study group (8.57%) was significantly lower than the control group (22.95%) (*p* < 0.05), as shown in Figure 1.

### Comparison of NIHSS and FMA Scores Between the Two Groups

**Figure 2:**
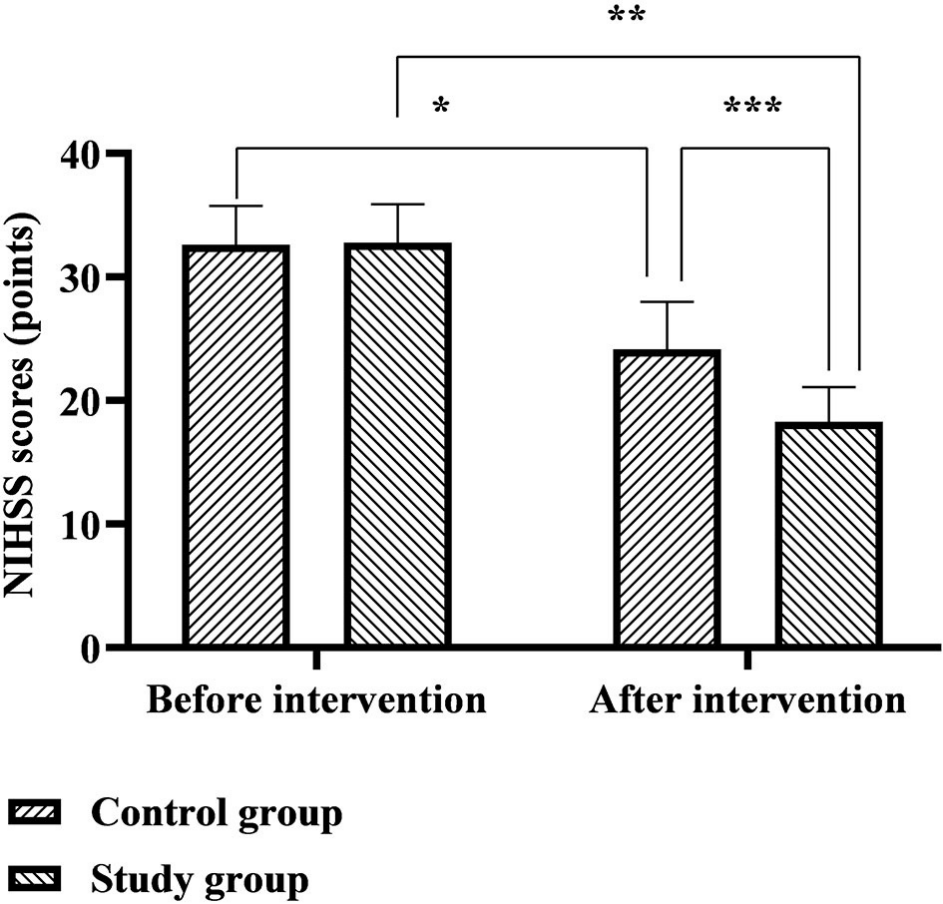
Comparison of NIHSS scores between the two groups. *Represents that there was a statistical difference in the NIHSS scores before and after the intervention in the control group (*t* = 13.390, *p* < 0.001); **represents that there was a statistical difference in the NIHSS scores before and after the intervention in the study group (*t* = 29.212, *p* < 0.001); ***represents that there was a statistical difference in the NIHSS scores of the control group and the study group after the intervention (*t* = 10.076, *p* < 0.001).

**Figure 3:**
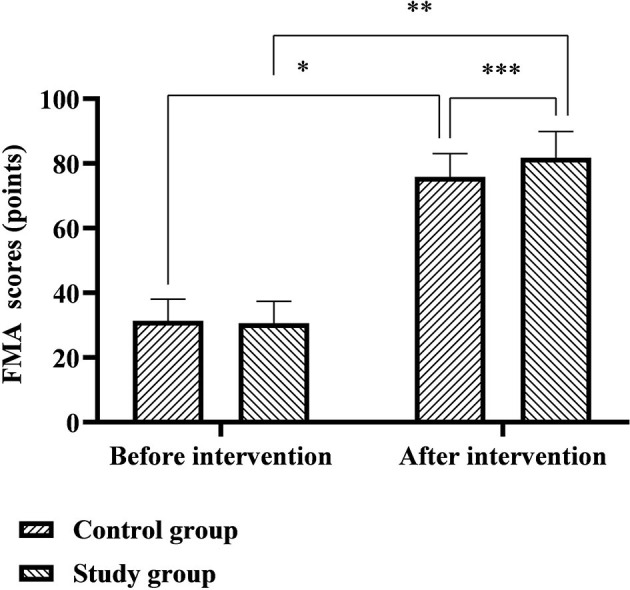
Comparison of FMA scores between the two groups. *Represents that there was a statistical difference in the FMA scores of the control group before and after the intervention (*t* = 35.630, *p* < 0.001); **represents that there was a statistical difference in the FMA scores of the study group before and after the intervention (*t* = 40.797, *p* < 0.001); ***represents that there was a statistical difference in the FMA scores of the control group and the study group after the intervention (*t* = 4.450, *p* < 0.001).

The NIHSS scores of the control group before and after the intervention were 32.62 ± 3.13 and 24.14 ± 3.83 points, respectively, and the NIHSS scores of the study group before and after the intervention were 32.80 ± 3.08 and 18.29 ± 2.79 points, respectively. The FMA scores of the control group before and after the intervention were 31.41 ± 6.63 and 75.86 ± 7.14 points, respectively, and the FMA scores of the study group before and after the intervention were 30.65 ± 6.72 and 81.82 ± 8.06 points, respectively. Six months after discharge, the NIHSS scores of the two groups were significantly lower than before nursing, the FMA scores were significantly higher than before nursing, and the study group was significantly better than the control group (*p* < 0.05), as shown in Figures 2, 3.

### Comparison of SS-QOL and MBI Scores Between the Two Groups

**Figure 4:**
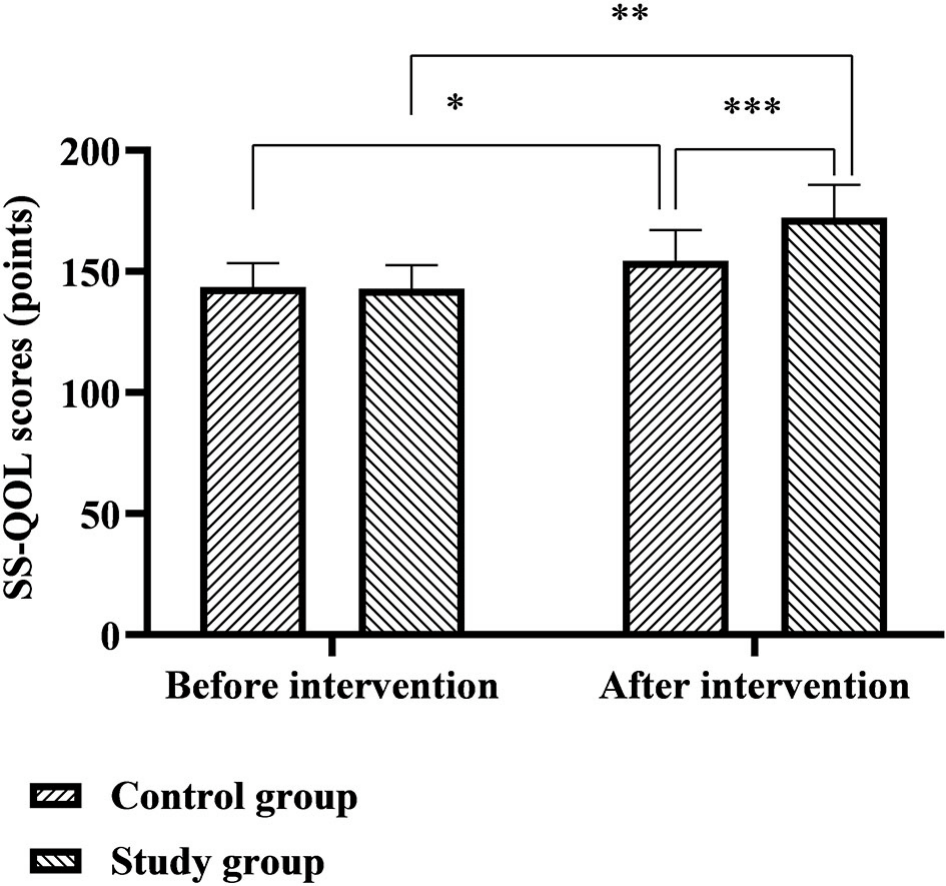
Comparison of SS-QOL scores between the two groups. *Represents that there was a statistical difference in the SS-QOL score of the control group before and after the intervention (*t* = 5.217, *p* < 0.001); **represents that there was a statistical difference in the SS-QOL score of the study group before and after the intervention (*t* = 14.713, *p* < 0.001); ***represents that there was a statistical difference in the SS-QOL scores of the control group and the study group after the intervention (*t* = 7.716, *p* < 0.001).

**Figure 5:**
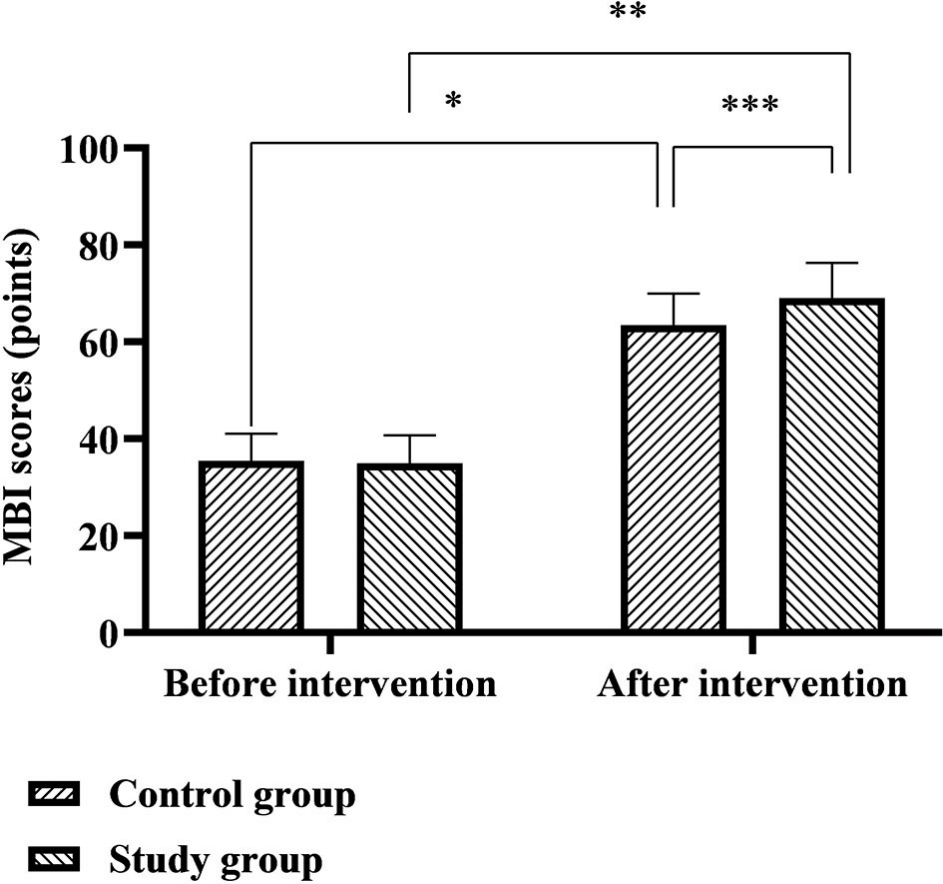
Comparison of MBI scores between the two groups. *Represents that there was a statistical difference in the MBI score of the control group before and after the intervention (*t* = 25.515, *p* < 0.001); **represents that there was a statistical difference in the MBI score of the study group before and after the intervention (*t* = 30.947, *p* < 0.001); ***represents that there was a statistical difference in the MBI scores of the control group and the study group after the intervention (*t* = 4.632, *p* < 0.001).

The SS-QOL scores of the control group before and after the intervention were 143.57 ± 9.83 and 154.34 ± 12.78 points, and the SS-QOL scores of the study group before and after the intervention were 142.89 ± 9.64 and 172.22 ± 13.61 points. The MBI scores of the control group before and after the intervention were 35.43 ± 5.61 and 63.48 ± 6.50 points, and the MBI scores of the study group before and after the intervention were 34.96 ± 5.77 and 69.06 ± 7.19 points. Six months after discharge, the SS-QOL and MBI scores of the two groups were significantly higher than before nursing, and the study group was significantly higher than the control group (*p* < 0.05), as shown in Figures 4, 5.

### Comparison of HAD-A and HAD-D Scores Between the Two Groups

**Figure 6:**
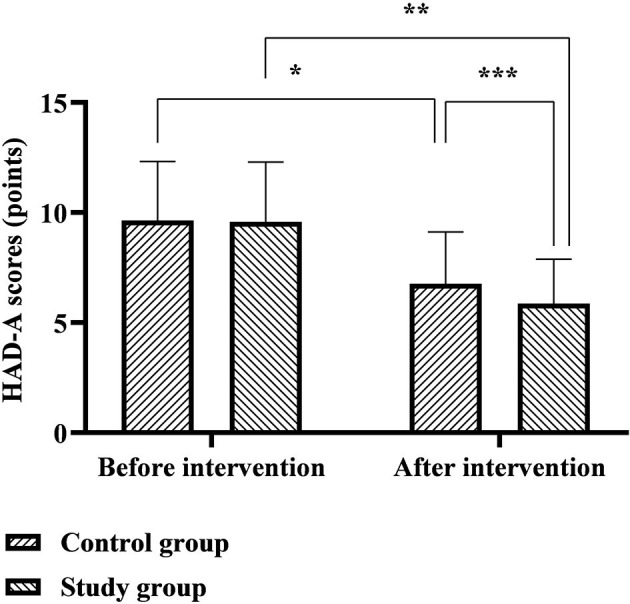
Comparison of HAD-A scores between the two groups. *Represents that there was a statistical difference in the HAD-A scores of the control group before and after the intervention (*t* = 6.289, *p* < 0.001); **represents that there was a statistical difference in the HAD-A scores of the study group before and after the intervention (*t* = 9.200, *p* < 0.001); ***represents that there was a statistical difference in the HAD-A scores of the control group and the study group after the intervention (*t* = 2.363, *p* < 0.05).

**Figure 7:**
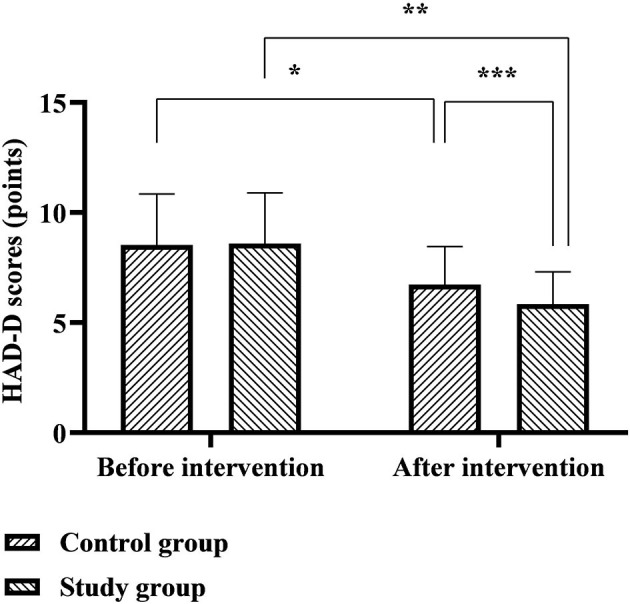
Comparison of HAD-D scores between the two groups. *Represents that there was a statistical difference in the HAD-D scores of the control group before and after the intervention (*t* = 4.868, *p* < 0.001); **represents that there was a statistical difference in the HAD-D scores of the study group before and after the intervention (*t* = 8.503, *p* < 0.001);***represents that there was a statistical difference in the HAD-D score between the control group and the study group after the intervention (*t* = 3.203, *p* < 0.05).

The HAD-A scores of the control group before and after the intervention were 9.64 ± 2.68 and 6.77 ± 2.35 points, and the HAD-A scores of the study group before and after the intervention were 9.58 ± 2.71 and 5.87 ± 2.01 points. The HAD-D scores of the control group before and after the intervention were 8.53 ± 2.32 and 6.73 ± 1.72 points, and the HAD-D scores of the study group before and after the intervention were 8.60 ± 2.29 and 5.84 ± 1.46 points. Six months after discharge, the HAD-A and HAD-D scores of the two groups were significantly lower than before nursing, and the study group was significantly lower than the control group (*p* < 0.05), as shown in Figures 6, 7.

### Comparison of Disability Rate and Readmission Rate Between the Two Groups

Six months after discharge, the disability rate of the study group (62.86%) was lower than that of the control group (75.41%), but there was no statistical difference between the two groups (*p* > 0.05). Within 6 months of discharge, the readmission rate of the study group (7.14%) was lower than that of the control group (18.03%), but there was no statistical difference between the two groups (*p* > 0.05), as shown in Table 2.

**Table 2:** Comparison of disability rate and readmission rate between the two groups [*n* (%)].

**Group**	***n***	**Degree of disability**						**Readmission rate**
		**Grade I**	**Grade II**	**Grade III**	**Grade IV**	**Grade V**	**Disability rate**	
Control group	61	1 (1.64)	2 (3.28)	10 (16.39)	34 (55.74)	14 (22.95)	46 (75.41)	11 (18.03)
Study group	70	1 (1.43)	1 (1.43)	9 (12.86)	34 (48.57)	25 (35.71)	44 (62.86)	5 (7.14)
*χ^2^*							2.389	3.605
*P*							0.122	0.058

### Comparison of Nursing Satisfaction Rate Between the Two Groups

The nursing satisfaction rate of the study group (98.57%) was significantly higher than that of the control group (88.52%) (*p* < 0.05), as shown in Table 3.

**Table 3:** Comparison of nursing satisfaction rate between the two groups [*n* (%)].

**Group**	***n***	**Very satisfied**	**Basically satisfied**	**Dissatisfied**	**Satisfaction rate**
Control group	61	26 (42.62)	28 (45.90)	7 (11.48)	54 (88.52)
Study group	70	57 (81.43)	12 (17.14)	1 (1.43)	69 (98.57)
*χ^2^*					5.738
*P*					0.017

## Discussion

According to Kolcaba's comfort theory, when the disease deprives the integrity of the patient's body or deprives the normal functioning of the brain and body functions, the patient can have a strong demand for treatment and comfort due to all the current experience (10). But in the regular care mode, there is a gap between the patient's comfort needs and the caregivers meeting their needs, and there is a gap between the effect of hospital nursing and the effect of out-of-hospital nursing. Based on this, this study applied comfort nursing to the nursing management of patients with ICH during the hospitalization period and applied continuation nursing to the nursing management of patients with ICH during the rehabilitation period after discharge from the hospital. It aimed to help patients with ICH achieve the maximum effect of nursing and treatment.

(1). Comfort nursing combined with continuous nursing intervention can effectively prevent the occurrence of pressure sores and falls in patients with ICH. Pressure ulcers are the long-term compression of local skin or soft tissues and then a kind of ischemia, hypoxia, or malnutrition ulcer necrosis, and it is one of the common complications of patients with coma, hemiplegia, or postural restriction caused by ICH (11). Falls are more common in patients with impaired motor and cognitive function and are also an important mechanism of injury leading to ICH (12, 13). During the nursing period of this study, the incidence of pressure sores and falls in the study group were significantly lower than that in the control group. Analyzing the reasons, under the guidance of comfort nursing and continuous nursing, the application of antidecubitus air mattresses for patients in hospitals and homes can automatically change the patient's pressure position, avoid continuous pressure on local skin tissues, and assist in turning over and knocking back regularly, and massage the bones and joints, etc., which can effectively promote blood circulation and reduce the occurrence of necrosis (14). In addition, a wealth of antifall knowledge can enhance the patient's antifall awareness and improve the ability to respond to fall behavior (15). However, because patients with ICH often have different degrees of motor and cognitive dysfunction, in this study, researchers used health education before discharge, such as on-site teaching, video exercises, health manuals, WeChat push, etc. to strengthen patients' memory, which is conducive to the improvement of patients' fall prevention behavior.

(2). Comfort nursing combined with continuous nursing intervention after discharge can effectively improve the quality of life of patients with ICH. Impairment of neurological function is an important feature of ICH, which creates physical and language dysfunction in patients, severely impaired cognition and quality of life, and further promotes the generation of negative emotions such as anxiety, depression, pessimism, and world-weariness (16, 17). The results of this study showed that 6 months after discharge from the hospital, the FMA, SS-QOL, and MBI scores of the two groups of patients were significantly higher than before nursing, the NIHSS, HAD-A, and HAD-D scores were significantly lower than before nursing, and the study group both were significantly better than the control group. It is suggested that the effect of comfort nursing combined with continuous nursing intervention after discharge on patients' neurological function, motor function, self-care ability, and mental health level is significantly better than that of conventional nursing. Analyzing the reasons, in the comfort nursing of this study, based on the conventional bed rest management, combined with evidence-based nursing basis and expert advice to implement targeted bedtime guidance, it will help not only to avoid the occurrence of rebleeding in the acute phase, but also to cultivate the ability of self-care in early life for people with mild symptoms and signs. In the comfort management of the lying position, paying attention to the maintenance of the functional position of the affected limb during the acute phase of the patient's disease will help to provide conditions for the later rehabilitation training. During the recovery period after discharge from the hospital, the patients are guided one by one through multiple channels such as online WeChat and offline home visits to expand the rehabilitation plan and training content, which not only solves the problem of patients' difficulty in obtaining systematic knowledge of rehabilitation nursing at home and in the community, but also improves timeliness and effectiveness of communication between nurses and patients, which provides convenience for the patient's rehabilitation process. In addition, the comfort nursing in this study also focused on providing comfort interventions to patients from environmental, sleep, defecation, infusion, and psychological and social aspects. Among them, the warm and comfortable inpatient environment is conducive to the improvement of the patient's mood and the recovery of the disease; adequate and good sleep quality is conducive to the recovery of the patient's nerve function; comfortable defecation care is conducive to reducing the embarrassment of patients with defecation in bed; comfortable infusion care is conducive to alleviation patients suffering from pain and discomfort during infusion therapy; psychological comfort care can provide patients with psychological support and stimulate their self-recovery potential and self-exercise perseverance; social comfort care can help to create a positive recovery atmosphere and reduce patients' psychological barriers. It can be seen from the above that the two nursing models used in this study are effective models that fully meet the comfort needs and rehabilitation needs of patients from the physical, psychological, and social levels.

(3). Comfort nursing combined with continuous nursing intervention after discharge can improve the short-term prognosis of patients with ICH to a certain extent. ICH is a type of stroke related to the highest disability rate and the highest mortality rate, and active and effective functional rehabilitation training during the rehabilitation period is an important measure to improve the function of the patient's affected limb and reduce the degree of disability (18). However, the negative emotions caused by the disease and the cohesion and compliance of out-of-hospital nursing after discharge from the hospital are the important reasons that affect the completion of the rehabilitation training of patients (19, 20). Based on this, in the nursing design of this study, the positive psychological assistance to patients and the creation of a rehabilitation atmosphere run through the hospital and out-of-hospital nursing. In terms of realization form, through the communication and interaction between nurses and patients, nurses can timely understand the patient's psychological changes and the crux of the problem and provide symptomatic counseling and interpretation to improve the degree of cooperation in treatment; through the communication and mutual assistance between patients-to-patients, it is helpful to increase patients' recovery enthusiasm and social behavior, so that patients can consciously participate in rehabilitation activities. The results showed that the disability rate and readmission rate of the study group were lower than those of the control group, but there was no statistical difference between the two groups, suggesting that comfort nursing combined with continuous nursing intervention can reduce the patient's brain to a certain extent the degree of injury and reduce the readmission rate. In this study, the nursing satisfaction rate of the study group was significantly higher than that of the control group. It shows that the combination of comfort nursing and continuous nursing intervention improves patients' satisfaction with the nursing effect and has certain application prospects.

## Conclusion

To sum up, the combination of comfort nursing and continuous nursing intervention after discharge can effectively reduce the occurrence of pressure ulcers and falls during the care of patients with ICH and contribute to the improvement of their quality of life and prognosis. It is worthy of clinical promotion.

## Data Availability Statement

The original contributions presented in the study are included in the article/supplementary material, further inquiries can be directed to the corresponding author.

## Ethics Statement

The studies involving human participants were reviewed and approved by Xiangyang Central Hospital, Affiliated Hospital of Hubei University of Arts and Science. The patients/participants provided their written informed consent to participate in this study.

## Author Contributions

ZL is responsible for the collection of cases and the evaluation of relevant results. JW is responsible for the statistics of data and the writing of papers. HL is responsible for the guidance of the research process. All authors listed have made a substantial, direct, and intellectual contribution to the work and approved it for publication.

## Conflict of Interest

The authors declare that the research was conducted in the absence of any commercial or financial relationships that could be construed as a potential conflict of interest.

## Publisher's Note

All claims expressed in this article are solely those of the authors and do not necessarily represent those of their affiliated organizations, or those of the publisher, the editors and the reviewers. Any product that may be evaluated in this article, or claim that may be made by its manufacturer, is not guaranteed or endorsed by the publisher.
